# Wee1 Inhibition by MK-1775 Leads to Tumor Inhibition and Enhances Efficacy of Gemcitabine in Human Sarcomas

**DOI:** 10.1371/journal.pone.0057523

**Published:** 2013-03-08

**Authors:** Jenny M. Kreahling, Parastou Foroutan, Damon Reed, Gary Martinez, Tiffany Razabdouski, Marilyn M. Bui, Meera Raghavan, Douglas Letson, Robert J. Gillies, Soner Altiok

**Affiliations:** 1 Experimental Therapeutics Program, H. Lee Moffitt Cancer Center and Research Institute, Tampa, Florida, United States of America; 2 Department of Cancer Imaging Research, H. Lee Moffitt Cancer Center and Research Institute, Tampa, Florida, United States of America; 3 Department of Diagnostic Imaging and Sarcoma, H. Lee Moffitt Cancer Center and Research Institute, Tampa, Florida, United States of America; 4 Sarcoma Program, H. Lee Moffitt Cancer Center and Research Institute, Tampa, Florida, United States of America; 5 Department of Comparative Medicine, University of South Florida, Tampa, Florida, United States of America; University of Illinois at Chicago, United States of America

## Abstract

Sarcomas are rare and heterogeneous mesenchymal tumors affecting both pediatric and adult populations with more than 70 recognized histologies. Doxorubicin and ifosfamide have been the main course of therapy for treatment of sarcomas; however, the response rate to these therapies is about 10–20% in metastatic setting. Toxicity with the drug combination is high, response rates remain low, and improvement in overall survival, especially in the metastatic disease, remains negligible and new agents are needed. Wee1 is a critical component of the G2/M cell cycle checkpoint control and mediates cell cycle arrest by regulating the phosphorylation of CDC2. Inhibition of Wee1 by MK1775 has been reported to enhance the cytotoxic effect of DNA damaging agents in different types of carcinomas. In this study we investigated the therapeutic efficacy of MK1775 in various sarcoma cell lines, patient-derived tumor explants *ex vivo* and *in vivo* both alone and in combination with gemcitabine, which is frequently used in the treatment of sarcomas. Our data demonstrate that MK1775 treatment as a single agent at clinically relevant concentrations leads to unscheduled entry into mitosis and initiation of apoptotic cell death in all sarcomas tested. Additionally, MK1775 significantly enhances the cytotoxic effect of gemcitabine in sarcoma cells lines with different p53 mutational status. In patient-derived bone and soft tissue sarcoma samples we showed that MK1775 alone and in combination with gemcitabine causes significant apoptotic cell death. Magnetic resonance imaging (MRI) and histopathologic studies showed that MK1775 induces significant cell death and terminal differentiation in a patient-derived xenograft mouse model of osteosarcoma *in vivo*. Our results together with the high safety profile of MK1775 strongly suggest that this drug can be used as a potential therapeutic agent in the treatment of both adult as well as pediatric sarcoma patients.

## Introduction

Sarcomas comprise a large number of rare, histogenetically heterogeneous, mesenchymal tumors affecting both pediatric and adult populations [1]. Approximately 10% of childhood cancers and 8% of young adult cancers are sarcomas, compared to cancer incidence of 1% in people over 40 years of age. The treatment of sarcomas frequently involves utilization of multimodality therapy, including surgery and radiation therapy with or without traditional chemotherapy. There is no therapeutic regimen that is used across all types of sarcomas, but gemcitabine and docetaxel are frequently employed in the treatment of sarcomas of different histological types, particularly in the second-line setting. However, despite the availability of novel molecularly targeted drugs in recent years, the cure rates have only been very modestly improved for metastatic and recurrent sarcomas [2].

One of the well-characterized mechanisms of resistance to DNA damaging agents is induction of G2/M cell cycle arrest through inactivation of the CDC2-cyclin B complex, which allows tumor cells to repair and survive DNA damage upon treatment [3]. CHK1 and Wee1 kinases play important roles in the regulation of CDC2 activity where increased phosphorylation of the tyrosine-15 (Tyr15) residue maintains the CDC2-cyclin B complex in an inactive state [4]. In contrast, activation of CDC2 through removal of its inhibitory Tyr15 phosphorylation allows cells to enter the mitotic phase of the cell cycle [4]–[6], thus offering an attractive mechanism to overcome resistance to chemotherapy by forcing tumor cells with damaged DNA to enter into unscheduled mitosis and to undergo cell death, often referred to as mitotic catastrophe [7].

Several studies have demonstrated that pharmacological inhibition of Wee1 by the small molecule kinase inhibitor MK-1775 leads to removal of CDC2 phosphorylation at Tyr15 in tumor cells [8]. MK-1775 has been reported to induce anti-tumorigenic effects in different cancer types that harbor p53 mutations [9]–[12] when combined with cytotoxic agents. We have previously shown that MK-1775 has a cytotoxic effect on sarcomas as a single agent independent of p53 mutation status [13]. Here, we compared the therapeutic efficacy of MK-1775 alone and in combination with gemcitabine in different types of soft tissue and bone sarcomas.

## Materials and Methods

### Cell Culture and Experimental Treatments

MG63 (ATCC CRL-1427), A673 (ATCC CRL-1598), U2OS (ATCC HTB-96), and HT-1080 (ATCC CCL-121) cells (ATCC, Manassas, VA) were grown in Dulbecco’s modified Eagle’s medium supplemented with 10% fetal bovine serum, 1% (v/v) penicillin-streptomycin, and 1% (v/v) L-glutamine at 37°C in a 5% CO_2_ incubator. Stock solutions of the Wee1 inhibitor MK-1775 (Selleck Chemicals, Houston, TX) were dissolved in DMSO and added to the media at the indicated concentrations. Stock solutions of gemcitabine (Gemzar, gemcitabine HCL, Eli Lilly and Company, Indianapolis, IN) were dissolved in 0.9% sodium chloride. Control cells were treated with vehicle alone. Commercially obtained cells were not authenticated by the authors.

### Cell Growth and Viability Assays

Cells were treated with MK-1775 and gemcitabine alone or in combination at a constant ratio for 72 hours. Cell viability was measured by the CT-Blue assay (Promega). The combined effects of MK-1775 and gemcitabine were quantified using a combination index (CI) method developed by Chou and Talalay [14]. This method involves plotting dose-effect curves for each agent and their combination, using a median-effect equation: fa/fu = (D/Dm)*m*, where D is dose of drug, Dm is dose required for a 50% effect (equivalent to IC50), fa and fu are affected and unaffected fractions, respectively (fa = 1−fu), and *m* is the exponent signifying the sigmoidicity of the dose-effect curve. The computer software Xlfit version 4.3.1 (ID Business Solutions) was used to calculate the values of Dm and *m*. The CI used for the analysis of the drug combinations was determined by the isobologram equation for mutually nonexclusive drugs that have different modes of action: CI = (D)1/(D*x*)1+ (D)2/(D*x*)2+ [(D)1(D)2]/[(D*x*)1(D*x*)2], where (D)1 and (D)2 are relative concentrations of drugs 1 and 2 and *x* is the percentage of inhibition. CI <1, CI = 1, and CI >1 indicate synergism, additive effects, and antagonism, respectively.

### Western Blot Analysis

Both adherent and detached cells in tissue culture wells were collected in 15-mL conical tubes and centrifuged at 4°C for 5 minutes at 1000 rpm in an Eppendorf 5810R centrifuge. The supernatant was removed, and the cell pellet was rinsed with ice-cold PBS, after which ice-cold Universal Cell lysis buffer (Millipore, Billerica, MA) was added. Samples were sonicated, vortexed on ice every 10 minutes for 30 minutes, and then transferred to 1.5-mL microcentrifuge tubes and centrifuged for 10 minutes at 13,000 rpm at 4°C in an Eppendorf 5417R microcentrifuge. We used the Pierce BCA assay kit to determine protein concentrations, following manufacturer’s protocol (Thermo Fisher Scientific, Rockford, IL). Samples were heated to 95°C for 10 minutes prior to resolving on an SDS-PAGE using a 4–20% gradient gel (BioRad Industries, Hercules, CA) and transferred to a polyvinylidene difluoride membrane (Millipore) using a semi-dry transfer device (BioRad Industries). The membrane was blocked for 1 hour at room temperature in Pierce Superblock (Thermo Fisher Scientific) and probed for various antibodies. Enhanced chemiluminescent detection was performed following manufacturer’s protocols (Thermo Fisher Scientific).

### Antibodies

Rabbit γH2AX, CDC2-phosphorylated Tyr15, Cleaved Caspase 3, cyclin A and total poly(ADP-ribose) polymerase (tPARP) antibodies were purchased from Cell Signaling Technology (Watertown, MA). Mouse β-actin antibody was purchased from Sigma Aldrich Corp. (St. Louis, MO). Rabbit γH2AX for immunohistochemistry was purchased from Novus Biologicals (Littleton, CO). Cyclin A for immunohistochemistry was purchased from Thermo Fisher Scientific (Pittsburgh, PA).

### Patient-derived *ex vivo* Studies

The *ex viv*o assays were performed as previously described [15]. Briefly, tumor explants were exposed to vehicle, MK-1775 (500 nM), gemcitabine (3 µM), or a combination of MK-1775 and gemcitabine for 18 hours, and tissue fragments were collected for Western blot and morphological studies.

### *In vivo* Patient-derived Xenograft Osteosarcoma Experiments

All experimental protocols were approved by the Institutional Animal Care and Use Committee (IACUC) and Institutional Review Board at the University of South Florida. All animals were maintained and evaluated in accordance with IACUC standards of care in pathogen-free rooms at the H. Lee Moffitt Cancer Center (Tampa, FL). Fresh tissue was obtained from a chemotherapy-naïve, 52-year-old osteosarcoma patient at time of initial core biopsy of a distal femur osteosarcoma, which was metastatic to the lungs at the time of presentation. The patient provided written informed consent. The tumor was implanted subcutaneously into the flanks of 6-week-old female SHO/SCID athymic mice (Charles River Laboratory). When tumors reached a volume of 500 mm^3^, mice were individually identified and randomly assigned to treatment groups of 4 mice (6–8 evaluable tumors) in each group: *1*) control; *2*) MK-1775 (30 mg/kg p.o., twice daily on days 1, 3, 8, and 10); *3*) gemcitabine (100 mg/kg i.p., once daily on days 1, 3, 8, 10); or *4*) MK-1775 and gemcitabine in the above-mentioned doses. Drug doses and schedules were chosen based on the investigator brochure, findings of an ASCO abstract phase I preliminary study and prior studies [10], [16]. Tumor growth was evaluated twice per week by measurement of two perpendicular diameters of tumors with a digital caliper. Individual tumor volumes were calculated as volume = [(width)^2^ × length]/2. Relative change in tumor growth from day 0 (%) was calculated using the formula: [(dayX vol – day0 vol)/day0 vol]×100. Animals were sacrificed 4 days after the last dose of drug treatment, and tumors were harvested for histologic analysis.

### MRI Protocol

MRI and measurements were conducted within our institutional vivarium H. Lee Moffitt Cancer Center. Before imaging studies were started, animals were placed in an induction chamber and anesthetized using 2% isoflurane in medical grade O_2_. After complete anesthetic induction, the animals were restrained in a mouse cradle, part of an insertion device, and placed within the RF coil of the magnet while continuously receiving isoflurane. Respiratory function and temperature of the animals were monitored and controlled throughout the imaging session using the SAII System (Small Animal Instruments, Inc., Stony Brook, NY). Temperature control of the animals was achieved by a variable temperature gas unit and set to maintain a body temperature of 36±1°C.

All MR data were acquired using a 7-T horizontal magnet (ASR 310, Agilent Technologies) equipped with nested 205/120/HDS gradient insert and a bore size of 310 mm. Temperature control of the imaging gradients was achieved by means of a water chiller (Nestle Waters) and maintained at 12°C for all acquisitions. Using a 72-mm quadrature birdcage coil (Agilent Technologies), axial T_2_-weighted fast spin-echo (FSE) sequences were acquired (TE/TR = 60/1403 ms) with a resolution of 136 µm over 6 minutes. Applying the same axial slice plane and spatial resolution, a diffusion weighted sequence using four b-values (50, 500, 1000, and 2000) and TE/TR = 36/1881 ms also was run over 12 minutes.

Image reconstruction and volumetric analysis were performed in VnmrJ (Agilent Technologies). Tumor volumes were obtained from the high-resolution T_2_-weighted FSEs and measured by manually drawn regions of interest encompassing the entire tumors (VnmrJ).

### Histopathology and Immunohistochemistry

Postmortem examinations included visual inspection for metastatic disease in the liver, lung, and abdominal/pelvic lymph nodes. At necropsy, the primary xenograft tumors were collected for each animal. The tissues were stored in 10% neutral buffered formalin, until processing within 48 hours. Tissues were processed and embedded in paraffin, and 4- to 5-µm slices of the tissues were obtained using standard histological procedures. Slides were stained with hematoxylin and eosin (H&E) using standard histologic techniques and examined by two pathologists (SA and MB). For immunohistochemistry, slides were stained using a Ventana Discovery XT automated system (Ventana Medical Systems, Tucson, AZ) as per manufacturer's protocol with proprietary reagents. Slides were deparaffinized on the automated system with EZ Prep solution (Ventana). For Cleaved Caspase 3 and Cyclin A, heat-induced antigen retrieval method was used in Cell Conditioning 1 (Ventana). Primary antibody was used at a 1∶400 (for Cleaved Caspase 3) and 1∶200 (for Cyclin A) concentration in Dako antibody diluent (Carpenteria, CA) and incubated for 60 min. Rabbit secondary antibody (Ventana OmniMap) was incubated for 16 minutes. For γH2AX, a heat-induced antigen retrieval method was used in RiboCC (Ventana). Primary antibody was used at a 1∶50 concentration in Dako antibody diluent and incubated for 32 minutes followed by rabbit secondary antibody for 20 minutes. The detection system used was the Ventana OmniMap kit, and slides were then counterstained with hematoxylin. Slides were then dehydrated and coverslipped as per normal laboratory protocol. Slides were evaluated under an ×20 objective and digitized using a color camera mounted to the microscope.

### Image Analysis

Histological slides of clinical osteosarcoma biopsy tissue and xenograft mouse tumors stained with H&E were scanned using the Aperio™ (Vista, CA) ScanScope XT with a 20×/0.8NA objective lens at a rate of 2 min per slide via Basler tri-linear-array.

Image analysis was performed using an Aperio Genie® v1 customized algorithm to first segment regions of viable tumor and bone formation in conjunction with the pathologist’s review for quality control. The percentage of area of bone formation (µm^2^) per viable tumor area (µm^2^) was calculated.

For quantification of immunostained cells by Ki-67, Caspase-3, Cyclin A and γH2AX, randomly selected high-power (×400) images from each graft were captured as described above. Ki-67–, caspase-3–, cyclin A or γH2AX- positive cells and total cells (positive+negative) were counted independently by two pathologists (SA and MB). The percentages positive cells were calculated using the formula: Index = number of positive cells×100/number of total cells.

### Statistical Analysis

In Figures with error bars, these represent standard error of the mean. Significance was analyzed using unpaired Student's *t*-test. The differences were considered significant when the *P* value was less than 0.05.

## Results and Discussion

### MK-1775 and Gemcitabine Show a Synergistic Cytotoxic Effect in Sarcoma Cells

**Figure 1:**
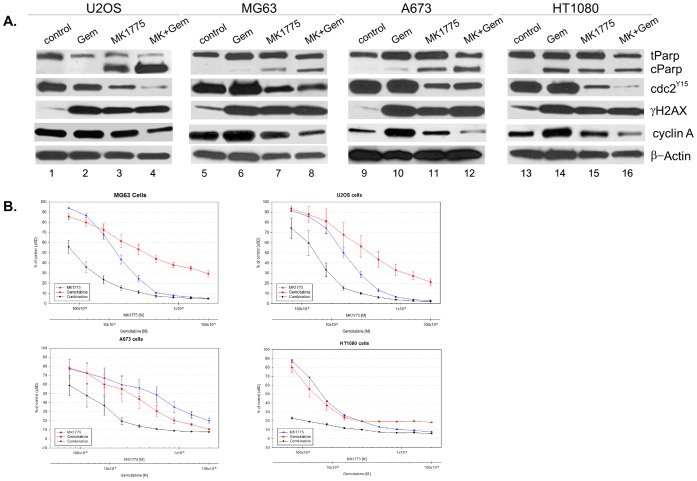
MK-1775 and gemcitabine show a synergistic effect in sarcoma cells. A. U2OS, MG63, A673, and HT1080 cells were treated with MK-1775 (500 nM) and gemcitabine (3 µM), either alone or in combination, for 24 hours, cell extracts were prepared, and Western blot analysis was performed with PARP (t: total, c: cleaved), CDC2-Tyr15, total CDC2, Cyclin A and γH2AX. β-Actin was used as a loading control. Experiments were repeated at least 3 times, and a representative experiment is shown. **B.** Cell viability assays of MG63, U20S, A673, and HT1080 cells treated with MK-1775 and gemcitabine, either alone or in combination, at a constant ratio for 72 hours. Drug concentrations are indicated on the horizontal axis and plotted against cell viability of control wells. Error bars represent ± SE of 4 replicate wells.

We have previously shown that MK-1775 as a single agent induces marked cell death in sarcoma cell lines [13]. To determine whether combination of MK-1775 with gemcitabine potentiates its cytotoxic effects in sarcoma cells, asynchronously growing MG63, U20S, A673, and HT1080 cell lines were treated with MK-1775 (500 nM) and gemcitabine (3 µM) at clinically relevant doses for 24 hours [8], and cell extracts were evaluated by Western blot analysis. To assess the activation of DNA damage response by MK-1775 and gemcitabine, we determined the phosphorylation status of histone H2AX (γH2AX) that occurs rapidly after genotoxic stress. We also analyzed drug-mediated changes in phosphorylation at Tyr15 to assess CDC2 activation and PARP cleavage as a means of assessing apoptotic cell death. As illustrated in Figure 1A, gemcitabine markedly induced γH2AX consistent with DNA damage, but resulted in only limited apoptotic cell death. In contrary, MK-1775 alone caused DNA damage with increased γH2AX expression and apoptosis. Interestingly, in cells treated with MK-1775 and gemcitabine together, there was increased apoptotic cell death. These findings suggest that combination treatment resulted in higher cytotoxic activity in sarcomas than each drug alone. It is unlikely that gemcitabine treatment simply shows its effect through G2/M arrest that is overcome by MK-1775 as there was no marked increase in CDC2 phosphorylation upon treatment of sarcoma cells with gemcitabine alone. Previous studies demonstrated that gemcitabine induces accumulation of different types of tumor cells in S-phase of cell cycle, which is accompanied by increased expression of Cyclin A [14]–[16]. To better understand the molecular mechanisms by which MK-1775 and gemcitabine interact on sarcoma cells we evaluated expression levels of Cyclin A, a well-known surrogate of S-phase entry, by western blot analysis. As illustrated in Figure 1A, gemcitabine treatment caused increased Cyclin A expression independent of p53 status, where the highest increase was observed in A673 and HT1080 cells. Interestingly, in all cell lines tested, Cyclin A expression was found to be lowest in the presence of MK1775 and gemcitabine combination compared to other treatment groups, which was accompanied by a simultaneous decrease in inhibitory phosphorylation of CDC2. No changes were observed in total CDC2 levels in drug-treated cells compared to vehicle-treated controls (data not shown). These results indicate that MK-1775 disrupts gemcitabine induced S-phase arrest and forces unscheduled mitotic entry of DNA-damaged cells by simultaneously abrogating the G2/M checkpoint control. Given the importance of Wee1 in the genomic stability and DNA replication in S-phase [17] these findings also speculate that Wee1 activity might be required for gemcitabine induced S-phase arrest upon DNA damage. Our results are supported by a recent study demonstrating that MK-1775 overcomes S-phase arrest in response to gemcitabine treatment in breast cancer cells [18].

To determine whether MK-1775 and gemcitabine have synergistic effects, we next performed median dose-effect analysis. For this purpose, all cell lines were exposed to increasing concentrations of MK-1775, gemcitabine, or the combination of these drugs in a fixed ratio. Cell viability was assessed using CT-Blue assay, and the effect was analyzed using median effect analysis. As shown in Figure 1B, exposure of sarcoma cells to the combination of MK-1775 and gemcitabine exerted a strong synergistic effect with CI values of 0.5, 0.2, 0.6, and 0.1 for U2OS, MG63, A673, and HT1080 cells, respectively, indicative of a synergistic to strong synergistic effect (CI range 0.1–0.3 = ++++strong synergism, 0.3–0.7 = +++synergism; Table 1).

**Table 1:** Combined effects of MK-1775 and gemcitabine were quantified with the Chou and Talalay combination index (CI) method [14].

cell line	tumor type	Synergy	p53 status
U2OS	osteo	+++	WT
MG63	osteo	++++	null
A673	Ewing	+++	mut
HT1080	fibro	++++	WT

The CI used for drug combination analyses was determined by the isobologram equation. Ranking symbols (+/−) indicate average calculated Chou and Talalay combination index (CI) range (+++, synergism; ++++, strong synergism).

Previous studies showed that pharmacological inhibition of Wee1 enhances the cytotoxic effect of DNA-damaging agents [7], [9]–[11], [19]–[21], particularly in cancer cells harboring p53 mutations, likely because p53-deficient cells are largely dependent on G_2_/M arrest to repair DNA damage caused by cytotoxic agents. Based on these findings, p53 mutation has been proposed as a predictor of tumor response to Wee1 inhibitors in clinical trials [7], [22]. Our recent data demonstrated that, in sarcoma cell lines and patient tumors, p53 status is not predictive of response to MK-1775 as a single agent [13]. As shown in Figure 1C, a synergy is observed between MK-1775 and gemcitabine in all cell lines independent of their p53 status.

### MK-1755 and Gemcitabine Induce Apoptotic Cell Death in Patient-derived Tumor Explants *ex vivo*

**Figure 2:**
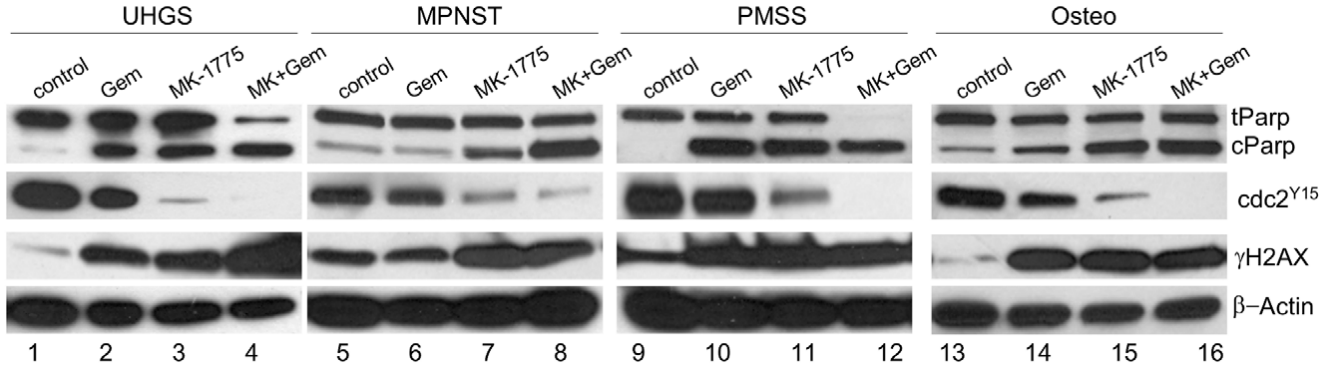
MK-1775 and gemcitabine combination treatment results in increased cleaved PARP and decreased CDC2 phosphorylation. Undifferentiated high-grade sarcoma (UHGS), malignant peripheral nerve sheath tumor (MPNST), pleomorphic spindle cell sarcoma (PSCS) and osteosarcoma (osteo) tumor explants were treated with MK-1775 (500 nM) either alone or in combination with gemcitabine (3 mM) for 24 hours. Cell extracts were prepared from tumor explants to assess expression levels of tPARP, CDC2^Y15^, and γH2AX by Western blot. β-Actin expression was used as loading control.

To evaluate MK-1775 and gemcitabine treatment in clinically relevant tumor samples, we tested the *ex vivo* effect of the combination treatment on patient derived tumors including undifferentiated high-grade sarcoma, malignant peripheral nerve sheath tumor, pleomorphic spindle cell sarcoma, and osteosarcoma (Figure 2). Tumor explants were treated with gemcitabine and MK-1775 for 24 hours and collected for Western blot analysis to assess target inhibition and cell death. As illustrated in Figure 2, compared to vehicle-treated control samples, *ex vivo* treatment of tumor explants with gemcitabine alone showed only limited cell death in all tumors, whereas MK-1775 (500 nM) caused marked cell death accompanied by increased DNA damage, and activation of CDC2, while no changes were observed in total CDC2 levels (data not shown). Consistent with the cell line data above, combination treatment with gemcitabine and MK-1775 had a higher cytotoxic effect on patient-derived tumor explants with increased PARP cleavage. Immunohistochemical analysis of p53 expression is commonly used as a surrogate for mutational analysis [23]–[26]. It has been generally accepted that wild-type p53 protein can barely be detected in normal cells by immunohistochemistry because it is normally a very short-lived protein, with a half-life of less than 30 minutes [27]. However, under conditions of DNA damage and other stresses, the wild type p53 level increases rapidly because of stabilization rather than increased synthesis that can be detected in normal cells. In contrast, mutant p53 has a longer half-life, and therefore, it accumulates in the nucleus and can be frequently detected by immunohistochemical studies [28], [29]. To determine whether p53 is mutated in patient-derived osteosarcoma, MPNST, PMSS and UHGS samples used in this study we performed immunohistochemical studies with a p53 antibody and showed that in all samples there were only occasional p53 positive cells (<1%) indicating the lack of p53 mutations in these tumors.

### MK-1775 as a Single Agent and in Combination with Gemcitabine Shows Antitumor Activity in Osteosarcoma *in vivo*

Osteosarcoma is the most common, non-hematopoietic, primary malignancy of bone consisting of abnormal osteoblastic cells with osteoid production [30]. Osteosarcoma is associated with poor prognosis due to its high incidence of metastasis and chemoresistance.

**Figure 3:**
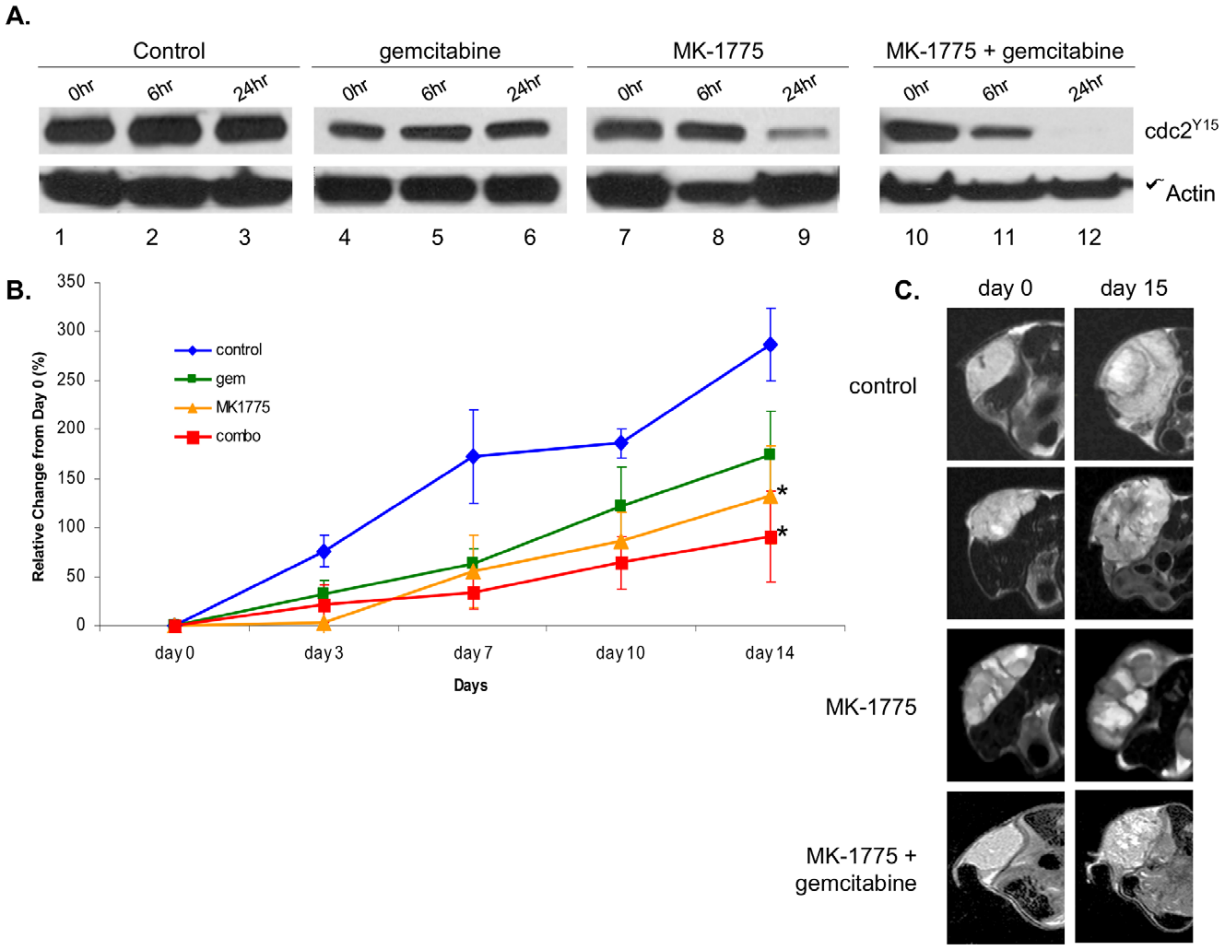
MK-1775 as a single agent and in combination with gemcitabine shows antitumor activity in human osteosarcoma xenografts. Animals were dosed with vehicle, gemcitabine, MK-1775, or a combination of gemcitabine and MK-1775 for 15 days, as described in Materials and Methods. **A.** FNA biopsies were taken before and 6 and 24 hours after treatment, and cell extracts were prepared to assess expression levels of CDC2^Y15^ by Western blot. β-Actin expression was used as loading control. **B.** Percent change from the initial tumor volume (i.e., day 0) as a function of time, displaying the significant increase in tumor growth from day 0 to 15 in the control group versus the other treatment groups. Treatments were administered on day 1, 3, 8, and 10; *indicates statistical significance (p<0.05). **C.** Representative T2-weighted MRIs show increased tumor volume at day 15 in vehicle and gemcitabine treatments compared to MK-1775-treated and combination-treated animals.

Wee1 has been reported to be over-expressed in various tumor types, including osteosarcomas, suggesting that it could play a role in chemoresistance and could be a potential therapeutic target in the treatment of this disease [31]. Here and in our previous studies [13], we showed that MK-1775 alone causes cell death in osteosarcoma cell lines at clinically achievable concentrations. To determine the *in vivo* efficacy of MK-1775 as a single agent and in combination with gemcitabine in the treatment of osteosarcoma, we used a xenograft mouse model of a p53 wild type osteosarcoma prepared with fresh tumor samples of a 52-year-old female who presented with a high grade osteosarcoma of the distal femur and numerous pulmonary metastatic nodules. The patient received two cycles of adriamycin and cisplatin with progressive disease and expired two months after the diagnosis. To determine the efficacy of MK-1775, gemcitabine, or combination of these drugs, xenograft mice were treated according to the clinical dose and schedule [25], [32] for 14 days. To predict and monitor the pharmacodynamic efficacy of drugs *in vivo*, tumor fine needle aspiration biopsy samples were obtained from the same tumor before and 6 and 24 hours after drug treatment, as previously described [33], and drug-mediated changes in the activity of CDC2-Tyr15 were tested by Western blot analysis. As illustrated in Figure 3A, treatment with MK-1775 alone and in combination with gemcitabine caused activation of CDC2 while no changes were observed in vehicle-treated control and gemcitabine groups demonstrating that MK-1775 effectively inhibits the activity of Wee1 at the early stage of treatment and gemcitabine does not interfere with its mode of action when combined *in vivo*.

Drug mediated changes in tumor volume were evaluated by measuring tumor size over the course of a 14 day treatment. As illustrated in Figure 3B, measurements revealed that tumors in the control group were larger in size than the tumors in any of the treated groups at all time points following treatment. For the groups receiving treatments, the data demonstrated similar tumor sizes and growth rate until day 9, at which time the tumors in vehicle-treated animals grew rapidly, whereas gemcitabine treatment resulted in approximately 40% inhibition of tumor growth and single-agent MK-1775 treatment resulted in approximately 50% inhibition of tumor growth. Combination of gemcitabine and MK-1775 caused an approximately 70% decrease in total tumor volume compared to control. Tumor inhibition in the combination group was higher than all other groups, suggesting that the combination treatment was better than gemcitabine or MK-1775 alone.

Diagnostic imaging methods such as MRI are important in evaluating the sarcoma’s response to chemotherapy. MRI can be used to differentiate solid tumor from necrosis and assess for the presence of fibrosis and calcification, all of which can be seen following treatment. Here, we used MRI to monitor drug-mediated changes in tumor content. The high-resolution T_2_-weighted FSE datasets allow for the detection and segmentation of tumor growth for all animals. Interestingly, as shown in Figure 3C, MRI analysis in MK-1775-treated animals and more so in combination groups revealed areas of very diminished (dark/black) signal, suggesting formation of calcification or was consistent with increased mineralization in tumors, where hypointense areas may be due to ossification.

**Figure 4:**
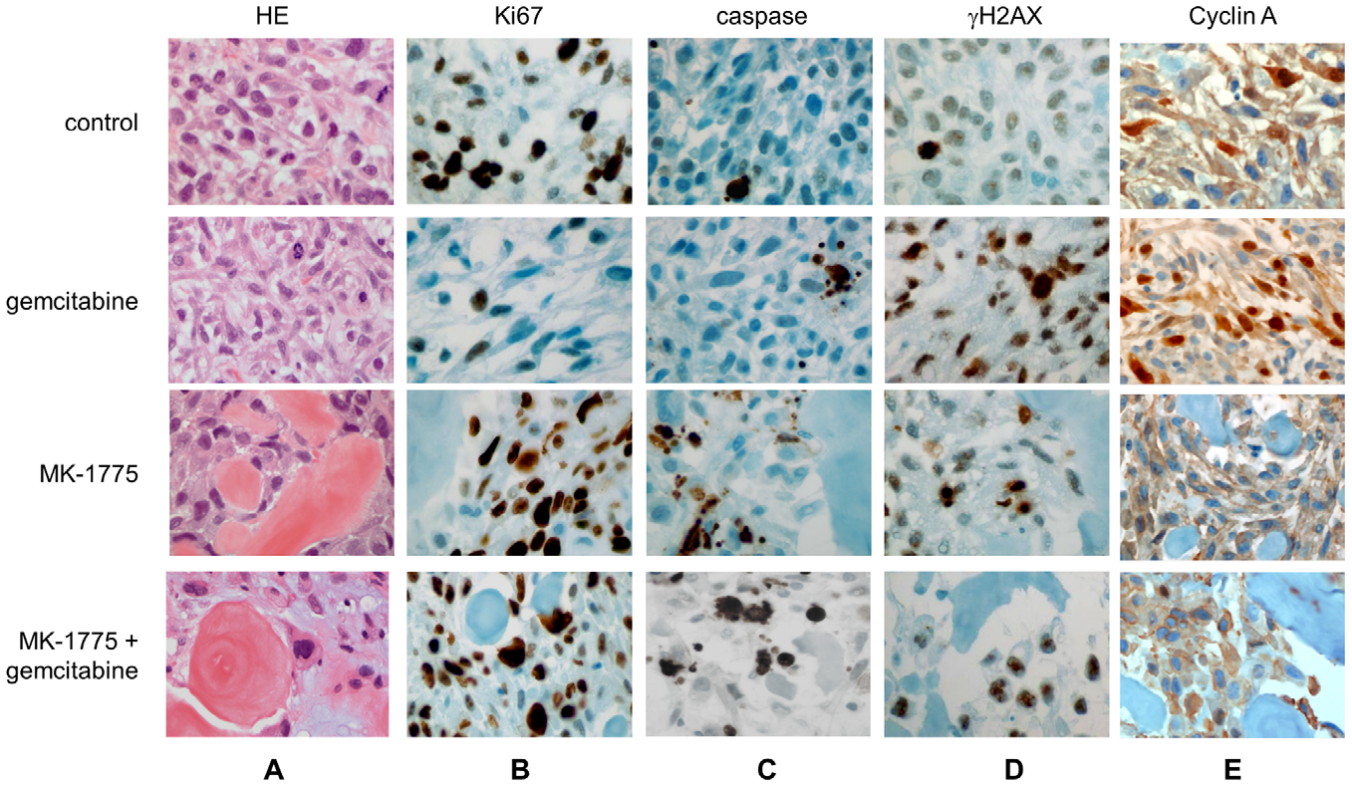
Histopathological assessment of tumor response to MK-1775 alone and in combination with gemcitabine in a patient-derived osteosarcoma mouse model. **A.** H&E-stained paraffin sections were prepared to assess microscopic features of cell death and differentiation representative micrographs of **B.** Ki67 shows high immunoreactivity in the control, MK-1775, and combination treatments compared to gemcitabine treatment. **C.** Cleaved-caspase 3 images show high immunoreactivity in the MK-1775 and combination treatments compared to vehicle and gemcitabine treatment. **D.** Slides stained for γH2AX show high immunoreactivity in gemcitabine, MK-1775, and combination treatments compared to controls. **E.** Slides stained for Cyclin A show higher immunoreactivity in gemcitabine but not in control, MK-1775 alone or in combination treatments.

To correlate imaging findings with the histological changes, tumors from control and treatment groups were collected on day 14 of treatment. Figure 4A shows that tumors in vehicle-treated control groups exhibited malignant spindle cells with moderate osteoid formation that is histologically similar to the patient’s tumor and consistent with high-grade osteosarcoma. The tumor samples obtained from the drug-treated groups had significant morphologic changes compared to the control samples. The most overt change was the amount of bone formation seen. In the control samples, the osteoid formation was approximately 10%, while it was 15% in gemcitabine-treated tumors, 45% in MK-1775-treated tumors, and 65% in gemcitabine+MK-1775-treated tumors. Additionally, the tumor cells in the MK-1775 alone and MK-1775+ gemcitabine groups exhibited increased cell size with abundant eosinophilic cytoplasm and significant osteoid production, consistent with differentiation (Figure 4B); results for the gemcitabine-treated group, however, were more similar to the those of the control group, where tumor cells were more spindled with scant cytoplasm.

As shown in our results and in our previous studies, MK-1775 treatment causes apoptotic cell death accompanied by increased mitotic activity and DNA damage [21]. To assess drug-mediated changes in the patient-derived osteosarcoma cells *in vivo*, immunohistochemical studies were performed with adequate controls after 14 days of treatment. As illustrated in Figure 4B, tumors treated with MK-1775 alone and with MK-1775+ gemcitabine had an increased Ki67 index of 73% and 79%, respectively, compared to tumors treated with gemcitabine and with vehicle (Ki67 index of 13% and 76%, respectively). The decreased Ki67 proliferation index in gemcitabine-treated tumors suggests that the decreased tumor growth observed in this group of mice is likely due to temporary cell cycle arrest activated by DNA damage, a mechanism that is frequently used by cancer cells to allow enough time to repair damaged DNA that contributes to drug resistance [34]. To evaluate drug-mediated cytotoxic effects, we performed immunohistochemical studies with cleaved caspase 3 antibody. In the vehicle-and gemcitabine-treated tumors, caspase activity was observed in 2% and 6% of tumor cells, respectively (Figure 4C). Interestingly, despite the lack of apoptotic cell death, gemcitabine-treated tumors revealed a significant increase (96%) in γH2AX immunoreactivity compared to the control groups (Figure 4D), demonstrating that treatment causes DNA damage but fails to induce cytotoxic response. As illustrated in Figure 4, combination of MK-1775 and gemcitabine caused an increased cytotoxic response, with 34% of cells showing cleaved caspase 3 that was accompanied by a Ki67 index of 79% and γH2AX index of 80%. These results show that MK-1775 is capable of overcoming gemcitabine-induced growth arrest and causes enhanced cytotoxicty in osteosarcoma cells *in vivo*. In the cell line studies above we showed that MK-1775 disrupts gemcitabine-induced S-phase arrest and leads to mitotic entry and cell death (Figure 1A). Next, we tested whether MK-1775 can overcome gemcitabine mediated S-phase arrest to enhance cell death in this patient-derived osteosarcoma model *in vivo*. To determine the population of cells in the S-phase of cell cycle we performed immunohistochemical studies with a Cyclin A antibody. As shown in panel E, compared to control group, gemcitabine treatment led to a 2-fold increase in the number of Cyclin A positive cells (13.5 vs 27.3%), while combination of MK-1775 with gemcitabine markedly decreased Cyclin A expressing cells (3%), which is similar to the MK-1775 treatment group (2.9%). These findings together with enhanced CDC2 activity are most consistent with a model where inhibition of Wee1 activity disrupts gemcitabine induced S-phase arrest and forces cells into mitosis with damaged DNA causing mitotic cell death.

Osteogenic differentiation is a complex process and is tightly regulated to ensure proper bone formation. Although certain genetic conditions and alterations increase the risk of developing osteosarcoma, the molecular pathogenesis is not well understood. However, defects in osteogenic differentiation have been linked to development of osteosarcoma [35], [36] while cellular immortalization attenuates osteoblastic differentiation [37], [38]. Hence, targeting the defects in osteogenic differentiation to promote terminal differentiation is an attractive approach in the treatment of osteosarcoma.

Several therapeutic interventions have been developed to target the differentiation process in different cancer types. For example, in breast cancer, tamoxifen induces differentiation and associated apoptosis by blocking estrogen receptor-mediated cellular proliferation [39], while in patients with prostate cancer, antiandrogens and retinoids can decrease tumorigenesis by promoting differentiation [40]. Similarly, ARA-C can induce differentiation and consequently complete remission in patients with acute myeloid leukemia [41], while all-trans retinoic acid leads to differentiation in neuroblastoma and in acute promyelocytic leukemia [42], [43].

Our data demonstrated that MK-1775 alone and in combination with gemcitabine induces significant terminal differentiation and cell death in high-grade osteosarcoma cells, laying an important foundation for future clinical trials with this well-tolerated readily available targeted drug.
